# Reliability and Accuracy of the Outerbridge Classification in Staging of Cartilage Defects

**DOI:** 10.1111/os.14016

**Published:** 2024-03-15

**Authors:** Moritz Mederake, Vivien Scheibe, Philipp Dalheimer, Daniel Schüll, Danalache Marina, Ulf Krister Hofmann

**Affiliations:** ^1^ Department of Trauma and Reconstructive Surgery, BG Klinik University of Tübingen Tübingen Germany; ^2^ Medical Faculty of the University of Tübingen Tübingen Germany; ^3^ Laboratory of Cell Biology, Department of Orthopaedic Surgery University Hospital of Tübingen Tübingen Germany; ^4^ Department Orthopedic Surgery University of Tübingen Tübingen Germany; ^5^ Orthopaedic Practice Nagold‐Riedt, Chan, Dalheimer Nagold Germany; ^6^ Department of Orthopaedic, Trauma, and Reconstructive Surgery RWTH Aachen University Hospital Aachen Germany

**Keywords:** Arthroscopy, Articular Cartilage, Histology, Osteoarthritis, Outerbridge Classification

## Abstract

**Objective:**

The decision on whether or not and how to treat a local cartilage defect is still made intraoperatively based on the visual presentation of the cartilage and findings from indentations with an arthroscopic probe. The treatment decision is then usually based on grading according to established classifications systems, which, therefore, need to have high reliability and accuracy. The aim of the present study was to evaluate the reliability and accuracy of the Outerbridge classification in staging cartilage defects.

**Methods:**

We performed an observer arthroscopic study using the Outerbridge classification on seven fresh‐frozen human cadaveric knees, which collectively exhibited nine cartilage defects. To evaluate accuracy, defect severity was verified through histological examination. Interrater and intrarater reliabilites were calculated using Cohen's kappa and the intra‐class correlation coefficient (ICC 3.1).

**Results:**

The interrater and intrarater reliability for the Outerbridge classification ranged from poor to substantial, with 0.24 ≤ κ ≤ 0.70 and κ = 0.55 to κ = 0.66, respectively. The accuracy evaluated by comparison with the histological examination was 63% overall. The erroneous evaluations were, however, still often at the discrimination of grade 2 and 3. We did not find any relationship between higher experience and accuracy or intraobserver reliability. Taken together, these results encourage surgeons to further use diagnostic arthroscopy for evaluating cartilage lesions. Nevertheless, especially in grade 2 and 3, deviations from the histology were observed. This is, however, the point where a decision is made on whether to surgically address the defect or not.

**Conclusion:**

Diagnostic arthroscopy is the standard for cartilage lesion assessment, yet interobserver reliability is fair to substantial. Caution is warranted in interpreting varied observer results. The accuracy of the “simpler” Outerbridge classification is insufficient compared to histological examinations, highlighting the need for improved techniques in guideline‐based intraoperative decision‐making.

## Graphical abstract

Intraoperative decisions regarding cartilage defects rely on visual assessments and arthroscopic probe findings. Applying the Outerbridge classification to fresh‐frozen knees showed fair to substantial reliability, 63% overall accuracy, and challenges in defect grading. The “simpler” Outerbridge's accuracy falls short of histological examinations, emphasizing the need for improved guideline‐based decision‐making techniques.

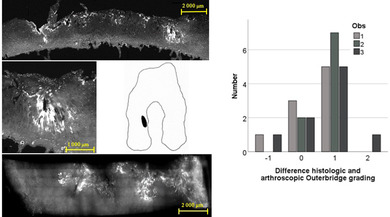

## Introduction

Osteoarthritis (OA) is one of the leading causes of pain and disability in adults across the globe.^1^ In the center of its physiopathology are the irreversible destruction and/or degeneration of the articular cartilage. While OA is defined as being a disease affecting the whole joint, in the initial stage, often isolated cartilage defects are present. Several classification systems have been established to grade the severity of such defects.^2^ As non‐invasive techniques to visualize the joint, X‐rays or magnetic resonance imaging (MRI) can be used. The former, however, only serve to grade higher stages of OA since the visualization of bone only gives indirect clues as to the condition of the cartilage. Modern MRI techniques allow, however, a good impression of both—severity and extent—of the cartilage defect.^3^, ^4^ Despite these advances, the decision of whether or not and how to treat a local cartilage defect is still made intraoperatively based on the visual presentation of the cartilage and findings from indentations with an arthroscopic probe. A modern system to classify these defects was proposed by the International Cartilage Regeneration and Joint Preservation Society (ICRS).^5^ While this system is scientifically well founded,^6^ it effectively requires the surgeon to correctly allocate the damage to one of 10 categories. The Outerbridge classification is much simpler (with a total of five different categories) and historically at the origin of the ICRS classification. Initially developed for chondromalacia patellae, it was later adapted to describe cartilage lesions throughout the joint^7^, ^8^, ^9^ and it is still commonly used by surgeons to grade intraoperative findings.^10^, ^11^, ^12^ The major determinant of the stage allocated is thereby the depth of the cartilage lesion with respect to the cartilage layer and the underlying bone (Table 1). From a clinical perspective, the key discrimination is between stage II (where mostly conservative treatment is recommended) and stage III (where indication for surgical cartilage regeneration attempts is given) (Table S1).

**TABLE 1:** Outerbridge classification and corresponding arthroscopic and histopathologic findings

Outerbridge Classification	Description	Arthroscopic findings	Histopathologic findings
0	Cartilage intact	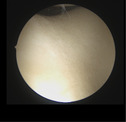	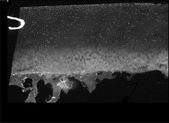
I	Superficial lesions	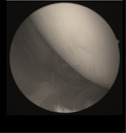	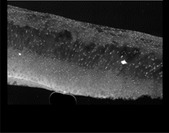
II	Lesions extending down to <50% of cartilage depth	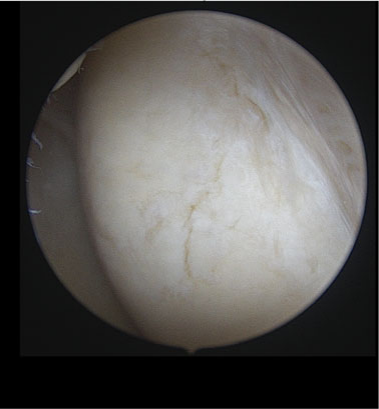	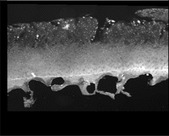
III	Defects extending down >50% of cartilage depth	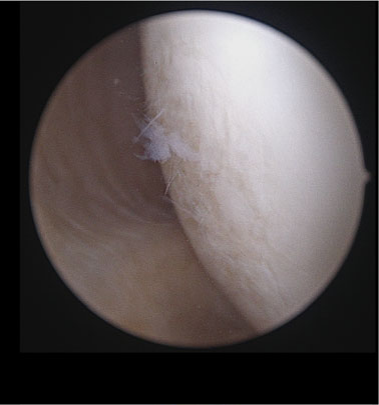	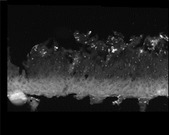
IV	Complete cartilage loss with defect through the subchondral bone	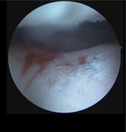	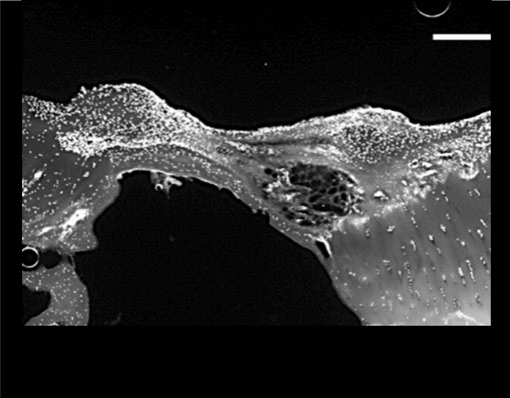

*Note*: The histologic images are displayed with the cartilage facing upward and the subchondral bone downward.

Despite its wide use in clinical practice, there is only a limited number of studies on the reproducibility and reliability of these systems. Regarding the Outerbridge classification, the κ coefficient in previous studies for interobserver reliability ranged from 0.28 to 0.52 and the κ coefficient for intraobserver reliability from 0.29 to 0.8.^13^, ^14^, ^15^, ^16^, ^17^ Despite the wide range of reliability, Cameron *et al*. also found that surgeons with greater experience had a higher level of reliability.^13^ Regarding the ICRS classification, there is only one study available that evaluates the reproducibility and, additionally, conducts a comparison with histological examinations. In that study, the interobserver and intraobserver reliability were 0.67 and 0.8, respectively. The correlation in depth with the histological results was 0.91.^18^ Of all studies mentioned, only one study actually verified the arthroscopic estimates using an additional technique, in this case by using a caliper for cartilage depth.^13^ A thorough verification of the classification system combining interobserver and intraobserver reliability assessments with histopathological examinations is still missing.

Using histopathologic evaluation of cartilage defects as a baseline, the aim of the present study was, thus, (i) to evaluate the precision of the arthroscopic Outerbridge classification and (ii) to measure its interobserver and intraobserver reliability.

## Materials and Methods

### Knee Specimen and Preparation Technique

Full departmental, institutional, and ethical approval were obtained before commencement of the study (registration number of the local ethical committee Nr. 009/2020BO2). Seven fresh‐frozen human cadaveric knees (Science Care, Phoenix, AZ, USA) were investigated, comprising a total of nine cartilage defects. The knees were obtained already separated from the donors at the middle of the thigh and the lower leg. Prior to the measurements, the specimens were thawed for 24 h in a water bath at room temperature. The mean age of the donors was 76 years (three male, four female). The mean body weight was 65 kg. Once thawed, soft tissues were removed from the diaphysis of the femur and the tibia/fibula to allow fixation for arthroscopy. A hole was drilled into the bone stumps, and a 6 × 40 mm screw was inserted. The bone ends including the screws were then cemented into resin blocks created using Technovit 2060 and Universal Liquid (Kulzer GmbH, Hanau, Germany) at a ratio of 2:1. Those cement blocks then served to fixate the femur and to move the tibia during arthroscopy.

### Arthroscopy Equipment

Arthroscopy equipment from Karl Storz, Tuttlingen, Germany was used to perform the arthroscopies and to acquire the images used for further grading. A 26″ full HD‐monitor visualized the findings during the procedure. Images and video sequences were registered by the documentation system Aida (Karl Storz). Rinsing of the joint with physiological saline was performed using the single flow mode of the Arthropump Power System (Karl Storz) at a pressure of 40 mmHg. A high‐flow shaft with a snap‐in shutter was used with the 0° and 30° Hopkins enlarged view, wide angle telescope. This setup was connected to the IMAGE1 S Connect camera platform. The cold light fountain D‐light C/AF SCB served as a light source (300‐watt xenon lamp). A standard setting for image acquisition was used with the combination of the parameters “standard”, “W”, and “white light”.

### Arthroscopic Defect Mapping

All arthroscopies were performed in a standardized fashion starting with an anterolateral portal and then a routine inspection of the joint. All possible cartilage defects were screened and registered. Relevant cartilage lesions were then separately addressed using such approaches that would best allow imaging. Altogether, the following portals were applied: anterolateral, anteromedial, superolateral, and accessory medial. The defects were then probed using a standard arthroscopy probe and photodocumented. The localization of the defects was detailed in a specifically drawn knee surface map to allow for later identification of the region of interest for histological sectioning (Figure S1).

### Cartilage Harvest and Histologic Imaging

**FIGURE 1:**
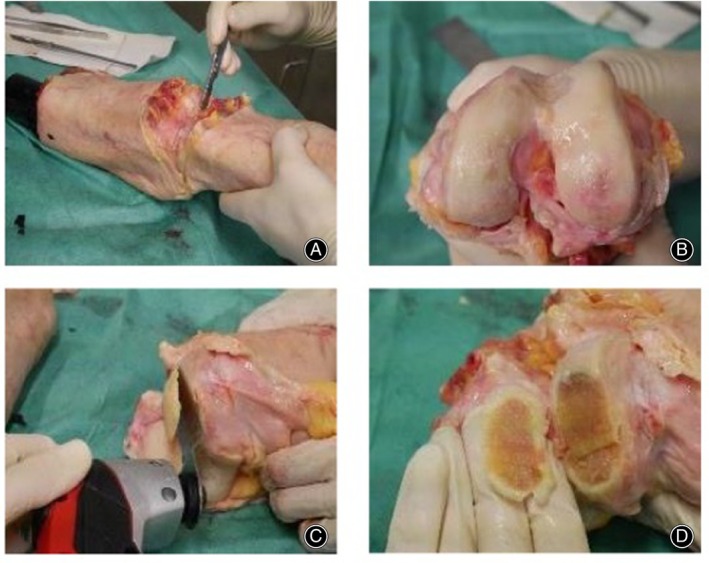
Cartilage harvest for histologic sectioning (with kind permission from Scheibe 2023).^28^ (A) Separation of the femur from the tibia at the joint line. (B) Femoral joint surface after removal of soft tissues around the joint. (C) Osteotomy of a bone cartilage segment containing the cartilage defect to be further analyzed. (D) Labelling of orientation by chipping out a triangle at one end of the bone cartilage segment.

After arthroscopy, the knee joint was freed from soft tissue to expose the articulating surfaces. Based on the mapping of the defects, these were reidentified und reconfirmed using the arthroscopy setup. Using an oscillating bone saw, the defects were resected *in toto*, including the underlying bone. The resected bone cartilage segments were labeled for orientation, then placed in phosphate buffered saline (PBS) and stored at 2–8°C until further processing (Figure 1).

After thawing at room temperature, specimens were fixated in 4% paraformaldehyde solution (pH 7.0) for 0.5–3 h at room temperature (21°C) depending on the sample size, with an estimated penetration speed of 1 mm from each side per hour. Decalcification was performed in 20% (w/v) ethylenediaminetetraacetic acid/PBS at 37°C for several days with solution changes every 2 days. Sufficient decalcification was determined by easy penetration of an 18‐gauge needle of the bone (7–14 days). DAPI (Exmax 358 nm, Emmax 461 nm, Life Technologies) fluorescent nuclear staining was then performed at a concentration of 0.1% (v/v)/PBS for 5 min. All images were acquired using a Carl Zeiss Observer Z1 fluorescence microscope (Carl Zeiss Microscopy). Top‐down views were performed to better visualize the defect. The site of the maximum cartilage defect was thus identified, and the tissue blocks labeled accordingly. Thereafter, the tissue block was cut with a cryotome blade at the site of the maximum defect. Then top‐down images were captured of one tissue block‐half as a whole with the AxioVision Release 4.8 software, using the MosaiX image acquisition packet (Carl Zeiss Microscopy, Jena, Germany). The other half of the tissue block was sectioned for side‐view analysis with a Leica cryotome, type CM1860 UV (Leica Biosystems, Wetzlar, Germany), at 35‐μm thickness. Mosaic images were then also taken of these side‐view images to visualize the depth of the defect.

The combination of top‐down and side‐view images was used to measure the size of the defect (width, length, and depth) and the corresponding Outerbridge grade was allocated.

### Evaluation of Cartilage Defects

Three independent observers (one senior experienced physician and two orthopaedic residents) evaluated the arthroscopic images. All evaluations and measurements were performed under identical conditions using the same computer, room, and light conditions. Grading was performed according to Outerbridge classification, and the maximum diameter of the defect was measured in pixels on the computer screen. Moreover, the presence of fissures extending from the main defect was registered in the following ordinal categories: no fissures, major fissure, side branches to a major fissure, and small side branches to the side branches.

Images were blinded and randomized before each evaluation round. Following the initial evaluation, images were again analyzed after a 4 weeks interval to evaluate intrarater reliability. Thereafter, all observers discussed arthroscopic images not belonging to the study images in a joint round to develop a more uniform understanding on how to classify cartilage defects on arthroscopic images. Thereafter, a third round of image evaluation was performed to obtain the best possible interrater reliability.

### Statistical Analyses

Interrater reliability was calculated on the results obtained from the second measurement round. Intrarater reliability was calculated separately for each observer between measurements 1 and 2. For the Outerbridge classification and the fissures, Cohen's kappa was calculated. Interrater reliability for defect size was evaluated with the intra‐class correlation coefficient (ICC 3.1) as a two‐way mixed form for consistency and absolute agreement. For interpretation of the obtained results, the recommendations from Cicchetti were applied, judging less than 0.40 as poor, 0.40–0.59 as fair, 0.60–0.74 as good, and 0.75–1.00 as excellent results. All reported *p*‐values are two sided with a significance level of α = 0.05. A graphic illustration was performed using boxplots. Statistical analysis was performed with SPSS Statistics 22 (IBM, Armonk, NY, USA).

To evaluate the accuracy of the arthroscopic Outerbridge grading, the ordinal difference between histologic and arthroscopic findings was calculated and the results displayed in the form of a bar diagram.

## Results

**FIGURE 2:**
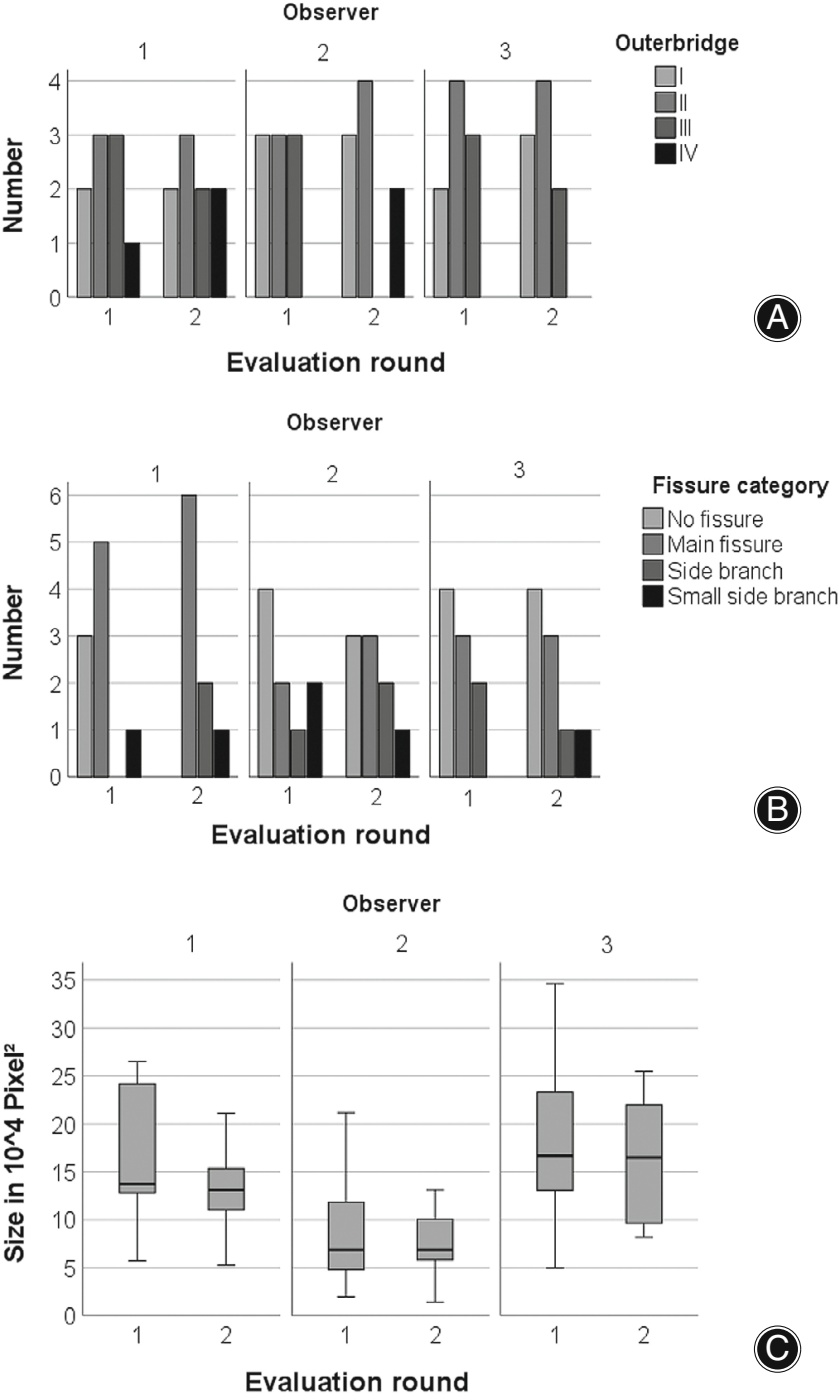
Interrater reliability. Bar diagrams showing the differences in Outerbridge grading of cartilage defects (A) and evaluation of fissures extending from the defect into the surrounding cartilage (B). Boxplots displaying the median of the maximum defect size measured (C) as evaluated on arthroscopic images. Interrater agreement for the Outerbridge classification and for the presence of fissures in the second measurement round ranged from poor to substantial (0.24 ≤ κ ≤ 0.70). Defect size agreement was good, with 0.731 (*p* = 0.002) for absolute agreement and 0.823 (*p* = 0.002) for consistency.

Interrater agreement for the Outerbridge classification and for the presence of fissures in the second measurement round ranged from poor to substantial (0.24 ≤ κ ≤ 0.70) (Table 2). The agreement between all observers for defect size as evaluated by the ICC was good, with 0.731 (*p* = 0.002) for absolute agreement and 0.823 (*p* = 0.002) for consistency (Figure 2).

**TABLE 2:** Interrater reliability for the Outerbridge classification and the presence of fissures between all observers

Grading	Observer 1/2	Observer 1/3	Observer 2/3
Outerbridge	κ = 0.70	κ = 0.24	κ = 0.36
	*p* < 0.001	*p* = 0.205	*p* = 0.059
Fissures	κ = 0.22	κ = 0.25	κ = 0.68
	*p* = 0.160	*p* = 0.069	*p* = 0.001

Abbreviation: κ, Cohen's kappa.

**FIGURE 3:**
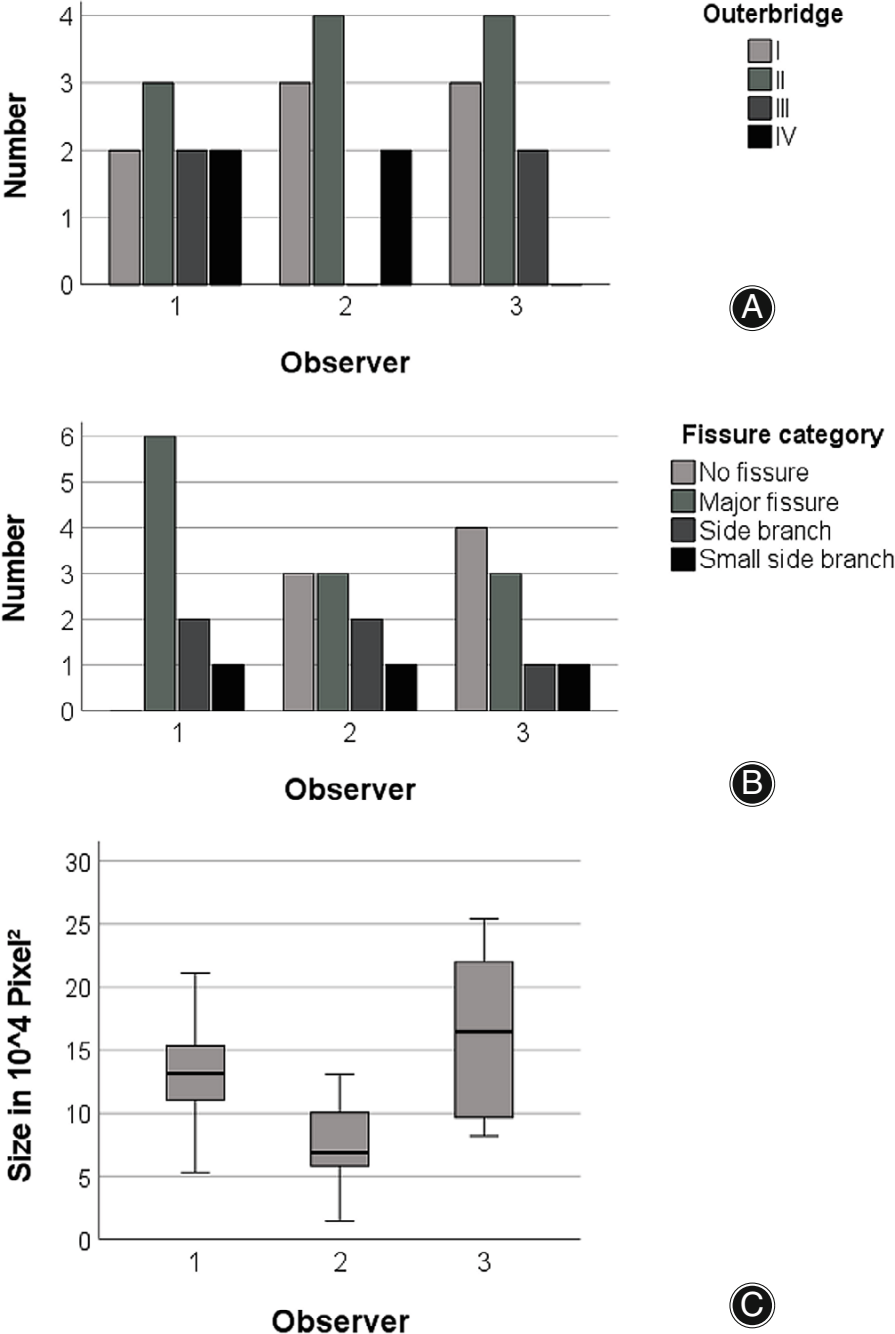
Intrarater reliability. Bar diagrams (A, B) and boxplots (C) showing the Outerbridge grading of cartilage defects (A), evaluation of fissures extending from the defect into the surrounding cartilage (B), and maximum size measured (C) as evaluated on arthroscopic images. The intrarater reliability reported here relates to the measurements between the first and second round for all three observers. Moderate to substantial agreement (κ = 0.55 to κ = 0.66) was found for the Outerbridge classification. For fissures, kappa ranged substantially from 0.10 to 0.83. Agreement for defect size was excellent (0.782 ≤ ICC ≤ 0.966).

The intrarater reliability as measured between the first and second round for all three observers showed a moderate or substantial agreement ranging from κ = 0.55 to κ = 0.66 for the Outerbridge classification. For fissures, an extreme range was observed, with a κ = 0.10 in one observer and an almost perfect agreement for another observer with a κ = 0.83. The agreement for defect size was excellent (0.782 ≤ ICC ≤ 0.966) (Figure 3, Table 3).

**TABLE 3:** Intrarater reliability for the Outerbridge classification, the presence of fissures, and defect size for all observers

Grading	Observer 1	Observer 2	Observer 3
Outerbridge	κ = 0.55	κ = 0.55	κ = 0.66
	*p* = 0.004	*p* = 0.001	*p* = 0.004
Fissures	κ = 0.10	κ = 0.39	κ = 0.83
	*p* = 0.495	*p* = 0.040	*p* < 0.001
Defect size—absolute agreement	ICC (3.1) = 0.90	ICC (3.1) = 0.97	ICC (3.1) = 0.78
	*p* = 0.002	*p* < 0.001	*p* = 0.024
Defect size—consistency	ICC (3.1) = 0.90	ICC (3.1) = 0.96	ICC (3.1) = 0.78
	*p* = 0.002	*p* < 0.001	*p* = 0.024

Abbreviations: ICC, intraclass correlation coefficient; κ, Cohen's kappa.

**FIGURE 4:**
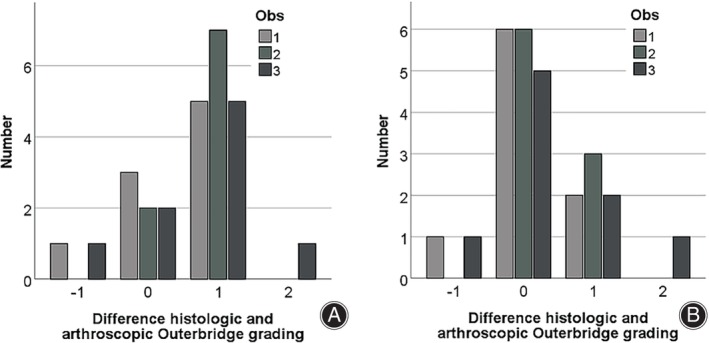
Accuracy of the Outerbridge grading and effect of training. Difference in grading between histologic and arthroscopic Outerbridge grading in three different observers calculated as ordinal values ranging from 0 to 4. (A) Accuracy in the first round of arthroscopic image evaluation. (B) Accuracy in the third round of arthroscopic image evaluation after a joint training session. A negative value means that arthroscopic grading overestimated defect severity, a positive value means underestimation. Abbreviation Obs‐observer.

In the histologic grading (Figures [Link], [Link], Table S1), 1 defect was classified as Outerbridge I, 3 as II, 2 as III, and 3 as IV. In the first read‐out round, only 7 out of 27 defects were correctly allocated, with the majority underestimating defect severity. In the third round, 17 out of 27 defects were correctly judged. In cases of erroneous stratification, again, defects were rather underestimated (n = 8) then overestimated (n = 2) (Figure 4, Table 4).

**TABLE 4:** Histologic and arthroscopic grading of nine different cartilage defects.

Histologic grading	Arthroscopic grading		
	Observer 1	Observer 2	Observer 3
2	2	2	2
3	2	2	2
2	3	2	3
3	2	2	2
1	1	1	1
4	4	4	4
2	2	2	2
4	4	3	2
4	4	4	4

## Discussion

In our prospective blinded study, the interrater and intrarater reliability for the Outerbridge classification ranged from poor to substantial with 0.24 ≤ κ ≤ 0.70 and κ = 0.55 to κ = 0.66, respectively. Accuracy evaluated by comparison with the histological examination was 63% overall. We did not find any relationship between higher experience and accuracy or intraobserver reliability. Of note, the erroneous evaluations were often at the discrimination of grade 2 and 3.

Arthroscopic evaluation is accepted as the standard of care in diagnosing and quantifying chondral lesions, especially in the knee.^19^, ^20^ However, beside factors such as size, depth, weight load and localization of lesions, the diagnostic value of this method is also affected by technical deliberations and the experience of the observer.^14^, ^16^, ^21^, ^22^, ^23^, ^24^ In the present study, arthroscopic pictures were evaluated by three different observers. One had little, one fair, and one much experience in diagnostic arthroscopies regarding size, depth (Outerbridge), and the presence of fissures. Beside the intraobserver and interobserver reliability, we also compared the visual gradings of the lesion depth with the histologically determined depth.

### Interobserver Reliability

The interobserver reliability in this study was poor to substantial for the Outerbridge classification (0.237 ≤ κ ≤ 0.695) as well as for the classification of fissures (0.224 ≤ κ ≤ 0.684) and good for the determination of defect size (0.731 ≤ ICC ≤0.823). An obvious reason for the only moderate reliability could be the difference in experience. However, in observer studies by Spahn *et al*., the reliability was even lower (0.127 ≤ κ ≤ 0.222; 0.052 ≤ κ ≤ 0.308), although the observers were highly experienced.^11^, ^25^ Different results were obtained by Dwyer *et al*. evaluating the ICRS classification. With an interobserver reliability of 0.67 and 0.8 for picture and video evaluation, respectively, they were able to present good reliabilities. Furthermore, these results suggest that the dynamic examination (video) is helpful in grading.^18^

### Intraobserver Reliability

Another key point for diagnostic methods is the reproducibility of results. We, therefore, calculated the intraobserver reliability for the first and second evaluation round. A wide range was seen for the classification of fissures with 0.10 ≤ κ ≤ 0.83. For the Outerbridge classification, results were moderate to substantial (0.55 ≤ κ ≤ 0.66). Interestingly, the most experienced observer presented the worst accordance. Adverse findings were presented by Lasmar *et al*. demonstrating a higher intraobserver reliability in more experienced surgeons compared to less experienced surgeons (κ = 0.50 *vs* −0.06).^15^ Other comparable studies showed results ranging from κ = 0.54 to κ = 0.8.^13^, ^14^ However, the results of the more experienced surgeons in these studies were also only moderate to substantial for the Outerbridge classification. Keeping this in mind, more detailed classification systems like the ICRS must be used with caution because the more classification possibilities there are, the lower the reproducibility.

### Accuracy

Of note, more important than the intraobserver and interobserver reliability of a diagnostic tool is the accuracy in determining the actual defect. Therefore, we used cadaveric knees and examined the defects histologically after arthroscopy. The comparison of accuracy was performed between the histologic grading and both the first and the third read‐out of each observer. The first comparison served as a status evaluation and the third to evaluate the best possible accuracy after a joint consensus meeting and to illustrate the effect of training. In the first round, less than a one‐third of evaluations actually matched the results obtained from the histologic evaluation. For most aberrant judgments, the severity of the defect was underestimated by one grade. In the third round, results had much improved, with only approximately one0third of erroneous evaluations. These were, however, still often at the discrimination of grade 2 and 3. To the best of our knowledge, there is only one comparable study evaluating the arthroscopically classified defects additionally histologically. Dwyer *et al*.^18^ The authors, also found a high correlation (0.91) of the arthroscopically and histologically grading. This seems to be true only for the main categories because the reliability decreases in the subgrades. However, contrastingly to our results, these surgeons tended to overrate the defect depth.^18^ When looking at the differences reported in the literature, an important aspect that might explain this phenomenon is the method of training of the observers, especially with respect to homogenization between observers. In the third round of measurements in our study (after the joint training session to homogenize measurements after the second round), all agreements for the Outerbridge classification were good or excellent (κ = 0.64; 0.66; 0.83) (Table S2). We had already observed a similar phenomenon when comparing the agreement between two spine surgeons and a trained medical student in the analysis of MRI for spinal stenosis.^26^

Taken together, these results should encourage surgeons to further use diagnostic arthroscopy for evaluating cartilage lesions. Nevertheless, especially in grade 2 and 3, deviations from the histology were observed. This is, however, the point where a decision is made of whether to surgically address a defect or not. Better techniques are, therefore, still warranted to further improve guideline‐based intraoperative decision‐making. Possible candidates for such evaluations could be, for example, micro‐ultrasound evaluations, optical coherence tomography‐based air‐jet indentation, or biomechanic testings, such as fiber Bragg grating (reviewed by Cykowska *et al*.^27^).

### Study Strengths and Limitations

In the present study we used a highly systematic approach applying strict criteria for data acquisition and processing. Performing the measurements with three independent observers, we were able to calculate both intraobserver and interobserver reliability. Performing mosaic imaging of histological sections, we were able to visualize the entire defects to evaluate their actual severity. The main limitation of the present study is the low number of investigated cartilage defects and the use of cadaveric specimens with partially osteoarthritic chondral lesions. However, already in this dataset, it becomes evident, that even the “simpler” Outerbridge classification lacks sufficient accuracy, considering that decision on treatment is based on such grading. It could be conjectured whether incorporating the option to utilize an arthroscopic probe could result in a considerably enhanced accuracy. Probing mostly helps in detecting softened cartilage and superficial lesions on one side (which is then a subspecification of grades 1 and 2) or to identify areas of delamination, which then equals a grade 4 defect. Probing does not, however, help to discriminate a grade 2 from a grade 3 defect. The histologic grading also might have underestimated but never overestimated the severity of the defect, if the area with the most pronounced lesion was not actually registered. In future projects, this problem might be addressed by using three‐dimensional imaging (e.g., μCT‐imaging).

## Conclusion

Diagnostic arthroscopy is still the standard of care for quantifying cartilage lesions. The interobserver reliability, however, seems to be only poor to substantial. Therefore, care must be taken when interpreting the results of other observers. Additionally, even the “simpler” Outerbridge classification is far from perfect in accuracy when comparing with histological examinations. Therefore, better techniques are warranted to further improve guideline‐based intraoperative decision‐making.

## Ethics Statement

The execution of this study adhered to the principles outlined in the Declaration of Helsinki. Departmental, institutional, and ethical approval were obtained before commencement of the study (registration number of the local ethical committee Nr. 009/2020BO2).

## Conflict of Interest Statement

Equipment for the arthroscopies was obtained from Karl Storz for this study only. Additionally, the company covered the expenses associated with obtaining cadaveric knees. All authors confirm that, apart from the funding provided for the presented study, they have no other conflicts of interest.

## Author Contributions

All authors listed meet the authorship criteria according to the latest guidelines of the International Committee of Medical Journal Editors, had full access to the data in the study, and take responsibility for the integrity of the data and the accuracy of the data analysis. M.M. designed and supervised the study, interpreted the arthroscopy images and also the final data, and wrote the manuscript; V.S. performed the tissue preparation, the histological experiments, and conducted the statistical analyses; P.D. co‐designed the study, performed the arthroscopies, and interpreted the arthroscopy images; D.S. helped with the arthroscopies and interpreted the arthroscopy images; M.D. helped with the histologic sectioning and interpreted the corresponding findings; U.K.H. co‐designed the study, performed the statistical analyses, and wrote the manuscript. All authors read and approved the final manuscript.

## Supporting information


**Figure S1.** Cartography of the knee articular surfaces to register the location of photodocumented cartilage defects to allow for their later identification for histologic sectioning. Femoral articulating surface on the left and tibial articulating surface on the right in a top‐down view.

**Figure S2.** Histologic Outerbridge grade I (with kind permission of Scheibe^28^). (A) Histologic section with DAPI nuclear staining for chondrocytes as a side view showing the still largely intact cartilage surface including the underlying bone with its trabeculae. (B) When enlarged, the erosion of the cartilage surface becomes visible. (C) Surface map of the femoral knee surface with the marked location of the cartilage defect. (D) Top‐down view of the corresponding cartilage block containing the defect.

**Figure S3.** Histologic Outerbridge grade II (with kind permission of Scheibe^28^). (A) Histologic section with DAPI nuclear staining for chondrocytes as a side view showing central defect in the cartilage layer that does not extend beyond 50% of the total cartilage thickness. (B) Showing the magnified defect illustrating that it remains restricted to the superficial and transitional zone. (C) Surface map of the femoral knee surface with the marked location of the cartilage defect. (D) Top‐down view of the corresponding cartilage block containing the defect.

**Figure S4.** Histologic Outerbridge grade III (with kind permission of Scheibe^28^). (A) Histologic section with DAPI nuclear staining for chondrocytes as a side view showing a pronounced cartilage defect with partially collapsing tissue and scar formation. The defect extends beyond 50% of the total cartilage but still respects the tide mark. The central maximum defect is surrounded by less but still relevantly compromised cartilage. (B) Showing the magnified defect with cluster and lacunae formation and a fibrous scar‐like tissue covering the defect. (C) Surface map of the femoral knee surface with the marked location of the cartilage defect. (D) Top‐down view of the corresponding cartilage block containing the defect.

**Figure S5.** Histologic Outerbridge grade IV (with kind permission of Scheibe^28^). (A) Histologic section with DAPI nuclear staining for chondrocytes as a side view showing a total loss in structural cartilage integrity with just scar tissue covering the subchondral bone and blister formation at the cartilage‐bone interface. (B) Maximum defect magnified. (C) Surface map of the femoral knee surface with the marked location of the cartilage defect. (D) Top‐down view of the corresponding cartilage block containing the defect.

**Table S1.** Recommended stage‐oriented cartilage therapy.^29^ “−” to “+++” degree of recommendation, with “−” being not recommended and “+++” being strongly recommended.
**Table S2.** Interrater reliability for the Outerbridge classification between all observers for the different measurement rounds. Joint training was performed after round 2. κ, Cohen's kappa.
