# Supplementary figures and images for: Reliability and Accuracy of the Outerbridge Classification in Staging of Cartilage Defects
Source: Orthop Surg. 2024 Mar 15;16(5):1187–95. doi: 10.1111/os.14016 (PMC11062859; doi:10.1111/os.14016)

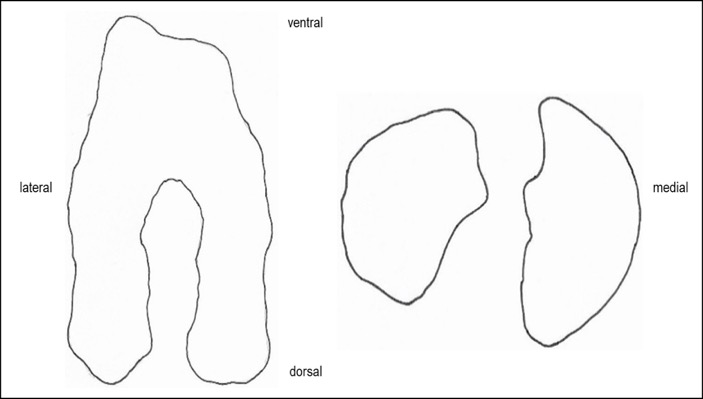

Supplement: Supplementary file 1 — Figure S1. Cartography of the knee articular surfaces to register the location of photodocumented cartilage defects to allow for their later identification for histologic sectioning. Femoral articulating surface on the left and tibial articulating surface on the right in a top‐down view. [file OS-16-1187-s001.jpg]

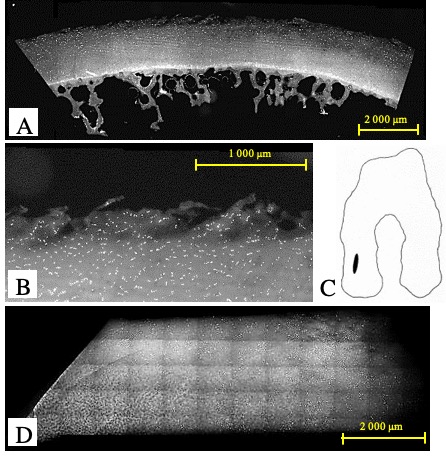

Supplement: Supplementary file 2 — Figure S2. Histologic Outerbridge grade I (with kind permission of Scheibe28). (A) Histologic section with DAPI nuclear staining for chondrocytes as a side view showing the still largely intact cartilage surface including the underlying bone with its trabeculae. (B) When enlarged, the erosion of the cartilage surface becomes visible. (C) Surface map of the femoral knee surface with the marked location of the cartilage defect. (D) Top‐down view of the corresponding cartilage block containing the defect. [file OS-16-1187-s004.jpg]

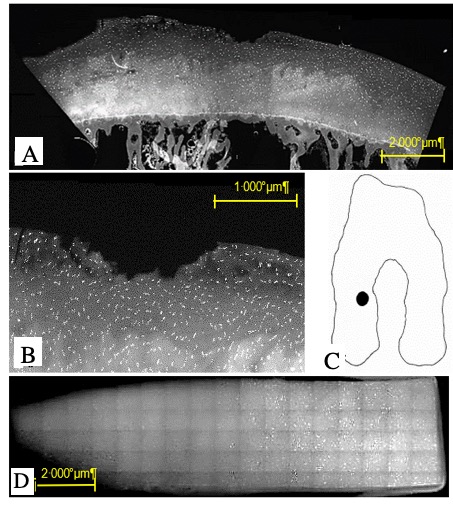

Supplement: Supplementary file 3 — Figure S3. Histologic Outerbridge grade II (with kind permission of Scheibe28). (A) Histologic section with DAPI nuclear staining for chondrocytes as a side view showing central defect in the cartilage layer that does not extend beyond 50% of the total cartilage thickness. (B) Showing the magnified defect illustrating that it remains restricted to the superficial and transitional zone. (C) Surface map of the femoral knee surface with the marked location of the cartilage defect. (D) Top‐down view of the corresponding cartilage block containing the defect. [file OS-16-1187-s003.jpg]

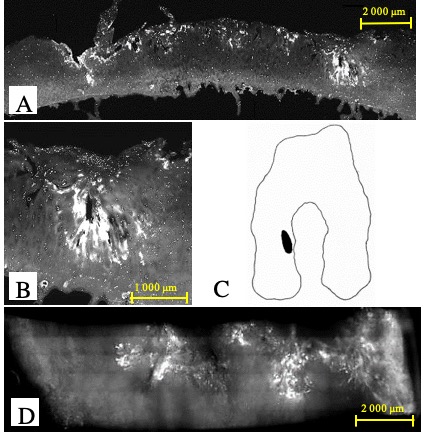

Supplement: Supplementary file 4 — Figure S4. Histologic Outerbridge grade III (with kind permission of Scheibe28). (A) Histologic section with DAPI nuclear staining for chondrocytes as a side view showing a pronounced cartilage defect with partially collapsing tissue and scar formation. The defect extends beyond 50% of the total cartilage but still respects the tide mark. The central maximum defect is surrounded by less but still relevantly compromised cartilage. (B) Showing the magnified defect with cluster and lacunae formation and a fibrous scar‐like tissue covering the defect. (C) Surface map of the femoral knee surface with the marked location of the cartilage defect. (D) Top‐down view of the corresponding cartilage block containing the defect. [file OS-16-1187-s006.jpg]

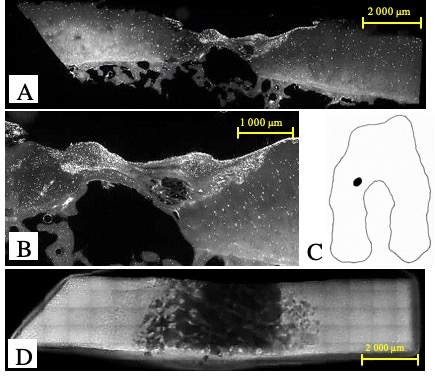

Supplement: Supplementary file 5 — Figure S5. Histologic Outerbridge grade IV (with kind permission of Scheibe28). (A) Histologic section with DAPI nuclear staining for chondrocytes as a side view showing a total loss in structural cartilage integrity with just scar tissue covering the subchondral bone and blister formation at the cartilage‐bone interface. (B) Maximum defect magnified. (C) Surface map of the femoral knee surface with the marked location of the cartilage defect. (D) Top‐down view of the corresponding cartilage block containing the defect. [file OS-16-1187-s005.jpg]
