# Supplementary material for: Reliability and Accuracy of the Outerbridge Classification in Staging of Cartilage Defects
Source: Orthop Surg. 2024 Mar 15;16(5):1187–95. doi: 10.1111/os.14016 (PMC11062859; doi:10.1111/os.14016)
Supplement: Supplementary file 6 — Table S1. Recommended stage‐oriented cartilage therapy. 29 “−” to “+++” degree of recommendation, with “−” being not recommended and “+++” being strongly recommended. Table S2. Interrater reliability for the Outerbridge classification between all observers for the different measurement rounds. Joint training was performed after round 2. κ, Cohen's kappa. [file OS-16-1187-s002.docx]

**Supplementary Tables**

**Supplementary Table 1:** Recommended stage oriented cartilage therapy (13). "–" to "+++" degree of recommendation with "-" being not recommended and "+++" being strongly recommended.

| **Outerbridge grade** | **Conservative treatment** | **Debridement** | **Microfracturing** | **(Mega-) OATS** | **ACT** |
| --- | --- | --- | --- | --- | --- |
| **I** | + | - | - | - | - |
| **II** | + | (+) | - | - | - |
| **III** | - | (+) | +++ | ++ | +++ |
| **IV** | - | (+) | - | +++ | ++ |

**Supplementary Table 2.** Interrater reliability for the Outerbridge classification between all observers for the different measurement rounds. Joint training was performed after round 2. κ: Cohen’s Kappa.

| **Grading Outerbridge** | **Observer 1/2** | **Observer 1/3** | **Observer 2/3** |
| --- | --- | --- | --- |
| **Round 1** | κ = 0.53  p < 0.012 | κ = 0.20  p = 0.353 | κ = 0.33  p = 0.150 |
| **Round 2** | κ = 0.70  p < 0.001 | κ = 0.24  p = 0.205 | κ = 0.36  p = 0.059 |
| **Round 3** | κ = 0.66  p = 0.002 | κ = 0.83  p < 0.001 | κ = 0.64  p = 0.003 |
